# Arteria lusoria dissection with mediastinal hematoma as a complication of a transradial coronary catheterization: Case report and literature review

**DOI:** 10.1016/j.ijscr.2020.09.146

**Published:** 2020-09-23

**Authors:** Raffaele Serra, Tiberio Rocca, Luca Traina, Noemi Licastro, Nicola Ielapi, Vincenzo Gasbarro

**Affiliations:** a Interuniversity Center of Phlebolymphology, International Research and Educational Program in Clinical and Experimental Biotechnology, University Magna Graecia of Catanzaro, Catanzaro, Italy; b Department of Medical and Surgical Science, University Magna Graecia of Catanzaro, Catanzaro, Italy; c Department of Morphology, Surgery and Experimental Medicine, University of Ferrara, Italy; d “Sapienza” University of Rome, Department of Public Health and Infectious Disease, Roma, Italy

**Keywords:** Arteria lusoria, Aberrant right subclavian artery, Mediastinal hematoma, Dissection, Endovascular, Transradial coronary catheterization

## Abstract

**Introduction:**

Aberrant right subclavian artery (ARSA), or arteria lusoria is the most common embrologic anomaly of the aortic arch. It is generally asymptomatic and incidentally diagnosed during a radiological exam or procedure.

**Presentation of case:**

Here, we report a case of ARSA incidentally diagnosed and injured (dissection with mediastinal hematoma) during a right transradial coronary angiography in a 83 years old female patient. The patient underwent prompt hybrid procedure with the isolation of the right humeral artery from where we positioned a GORE® VIABAHN® 9 × 100 mm endoprosthesis. The procedure was successful with optimal results at early and long term follow up.

**Discussion:**

Sometimes, a dissection of the ARSA may occur, especially for excessive manipulation during endovascular procedures, and when such complication happens it should be promptly treated as it can be life-threatening.

**Conclusion:**

If transradial catheterization during coronary angiography becomes particularly difficult, requires longer time, or the guide wire enters in the descending aorta, particularly attention should be paid, as dreadful complications such as dissection or lesion may happen and prompt treatment is required.

## Highlights

• Aberrant right subclavian artery (ARSA), or arteria lusoria is the most common embrologic anomaly of the aortic arch.
• The ARSA arises after the origin of the left subclavian artery and then reaches the posterior mediastinum.
• The presence of ARSA is generally asymptomatic, but may cause complications, during some endovascular procedures.
• Prompt diagnosis and treatment is required to avoid more dreadful complications.

## 1 Introduction

Aberrant right subclavian artery (ARSA), or arteria lusoria, first described by Hunauld in 1735, is one of the most common aortic arch anomalies which occurs in up to 2.44% of the general population but rises exponentially up to 34% in subjects with chromosomal defects such as Down syndrome [1,2].

In this condition, the ARSA arises after the origin of the left subclavian artery and then crosses upwards and to the right in the posterior mediastinum. The presence of ARSA is generally asymptomatic, but may cause difficulty as well as some complications, during some endovascular procedures, as in the case of right transradial access to perform coronary catheterization [[2], [3], [4]].

We present a case of ARSA incidentally diagnosed and injured during a right transradial coronary angiography providing a full revision of the current literature on this topic. This work has been reported in line with the SCARE criteria [5].

## 2 Case report

**Fig. 1:**
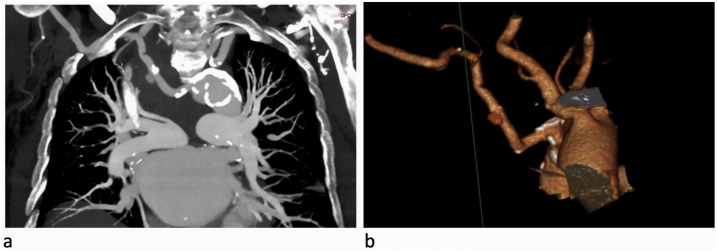
CT scan showing the ARSA and related pseudoaneurysm and the dissection (a); 3D CT reconstruction (b).

A female 83 years old patient presented with progressively worsening dyspnea started in the previous 3 days associated with orthopnea, and without chest pain. After initial cardiologic screening, and with an abnormal ejection fraction (EF) value, assessed with echocardiography (EF = 31%), the patient was admitted for coronary angiographic evaluation. A right radial approach was used, and nevertheless repeated attempts, catheterization resulted impossible, and then the procedure was stopped, as soon as the patient complained severe pain in the right hemithorax, and marked hypotension and bradycardia were also detected at the same time. A Computed Tomography Angiography (CTA) scan was therefore promptly performed that revealed the presence of ARSA, with pseudoaneurysm in the middle third of the artery with blood spreading in the upper posterior region of the mediastinum up to the carina of trachea, and also further downstream to the pseudoaneurysm, the presence of arterial dissection was also reported. It was also evident an abnormal origin of the right vertebral artery which raised from right common carotid artery (Fig. 1a-b).

**Fig. 2:**
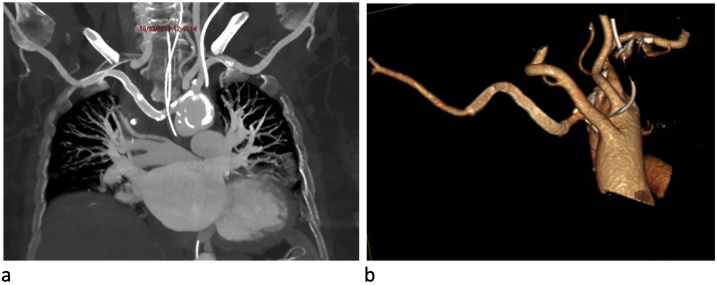
CT scan showing correct positioning of the endovascular stent graft (a); 3D CT reconstruction (b).

The patient was therefore promptly transferred to the operating room: after local anesthesia, surgical isolation of the right humeral artery, at the cubital fold, was performed, from where an 8Fr valve introducer was inserted, through which an X-ray Angiography (Xr-A) was performed to confirm and locate the ARSA lesion. Thereafter, we positioned a GORE® VIABAHN® 9 × 100 mm endoprosthesis for the exclusion of the pseudo aneurysm. After 24-hs, the CTA scan revealed correct positioning of the endovascular stentgraft, in correspondence with the lesion of the ARSA and the reported intimal flap slightly further downstream. There were no more evident signs of active bleeding, and the mediastinal hematoma of the upper-posterior mediastinum appeared reduced with a consensual reduction of the compressive effect on the trachea (Fig. 2a-b). After 4 months, the follow up CTA showed correct positioning of the stentgraft and complete resolution of the dissection. At duplex ultrasound of the right upper limb, good arterial circulation was also detected.

## 3 Discussion

The ARSA is caused by the irregular obliteration of the right fourth aortic arch during embryologic development. In some cases, it arises from the Kommerell’s diverticulum, a saccular outpocketing of the aorta, defined by an incomplete regression of the primitive right aortic arch [6,7].

The ARSA generally origins distal to the left subclavian artery and courses behind the esophagus or between this and the trachea or even anterior to this latter to reach the right arm. In doing so, the ARSA, especially if dilated, can cause dysphagia as reported in 1794 by Bayford that used the phrase “dysphagia lusus nature”, or dysphagia lusoria [8]. Nevertheless, this condition is often asymptomatic until right transradial coronary angiography is undertaken [2,3] and, in the context of an ARSA, only 60% of cases are successfully performed. In fact, more often the catheterization of the ascending aorta may be particularly difficult and takes longer time, or may be even impossible due to the acute angle between the arteria lusoria and the ascending aorta. In other circumstances the guide wire may even enters into the descending aorta, as the arteria lusoria is just adjacent to this segment [4]. In rare cases, a dissection and/or a lesion of the ARSA may occur, for excessive manipulation, as in the present case and, when such complications happen they should be promptly treated as they can be life-threatening. In fat, in our report, in consideration of the parietal lesion, the morphology and the trajectory of the ARSA, we placed a 9 mm wide, and 10 cm long GORE® VIABAHN® endoprosthesis, in order to stop bleeding and to ensure direct perfusion of the right subclavian artery. Using this stentgraft, we also have improved dissection stenosis in the middle third of ARSA that was located further downstream the parietal lesion. We have also extended the graft up to the passage of the artery from the thorax to the proximal part of the right upper limb to prevent any hemodynamically significant angles without covering also the right vertebral ostium, which, in this case, originated from the ipsilateral common carotid.

To our knowledge, this is first description of mediastinal hematoma caused by arteria lusoria dissection and injury following transradial coronary catheterization. Huang IL et al. [9] described a case of arterial lusoria dissection that resolved spontaneously with no specific treatment, due to the reduced extension of the dissection that didn’t determine hemodynamic alterations with the true lumen not compromised. Musuraca G et al. [10] described a dissection of arteria lusoria with arterial injury at its distal segment that determined right arm small and painful hematoma, and, even in this case, no treatment was performed because of the reduced extension of the dissection that resolved spontaneously. Hasan A et al. [3] described a dissection of arteria lusoria that was treated with conservative approach as the patient remained stable without signs of any deterioration of clinical parameters for 24 h. Therefore, it is evident that the management of ARSA dissection, which may be interventional or conservative, depends upon the clinical manifestations and the general and local clinical condition related to the patient.

In the other hand, ARSA aneurysmal degeneration may also occur, and this carries an important risk of rupture, therefore prompt diagnosis and treatment are mandatory. While there is no standardized treatment for this condition, and considering that open surgical repair is burdened by high mortality and complication rates, hybrid surgery based on a combination of supra-aortic vessel debranching and thoracic endovascular aortic repair or even total endovascular repair seem to be the best choices in terms of overall clinical success rate [[11], [12], [13]].

## 4 Conclusions

When transradial catheterization during coronary angiography becomes particularly difficult, requires longer time, or the guide wire enters in the descending aorta, particularly attention should be paid, as dreadful complications may happen. If a dissection, or an injury determining bleeding occurs, the treatment should be tailored around the general and clinical conditions of the patient, and the type and extension of the lesion. When conservative management is not indicated, hybrid surgery or even total endovascular approaches give the best results in terms of efficacy and safety.

## Declaration of Competing Interest

The Authors declare that there is no conflict of interest.

## Funding

This research received no specific grant from any funding agency in the public, commercial or not-for-profit sectors.

## Ethical approval

This study received exemption status by Institutional Review Board/Independent Ethics Committee of Interuniversity Center of Phlebolymphology (IRB/IEC – CIFL) at University Magna Graecia of Catanzaro. Exemption number: ER.FE.2018.32.EX.

## Consent

Written informed consent was obtained from the patient for her anonymized information to be published in this article.

## Author’s contribution

**Conceptualization:** Raffaele Serra and Vincenzo Gasbarro.

**Methodology:** Tiberio Rocca and Vincenzo Gasbarro.

**Acquisition of data:** Tiberio Rocca, Luca Traina, Noemi Licastro, and Nicola Ielapi.

**Analysis and interpretation of data:** Raffaele Serra, Nicola Ielapi, and Noemi Licastro.

**Writing - original draft preparation**: Raffaele Serra, and Nicola Ielapi.

**Writing - review and editing:** Tiberio Rocca, and Luca Traina.

**Supervision**: Raffaele Serra.

## Registration of research studies

N/A.

## Guarantor

Raffaele Serra and Vincenzo Gasbarro.

## Provenance and peer review

Not commissioned, externally peer-reviewed
